# Enhancing the Lap Shear Performance of Resistance-Welded GF/PP Thermoplastic Composite by Modifying Metal Heating Elements with Silane Coupling Agent

**DOI:** 10.3390/ma17204944

**Published:** 2024-10-10

**Authors:** Wanling Long, Xinyu Zhou, Bing Du, Xiangrong Cheng, Guiyang Su, Liming Chen

**Affiliations:** 1 Chongqing Key Laboratory of Nano-Micro Composite Materials and Devices, College of Materials and New Energy, Chongqing University of Science and Technology, Chongqing 401331, China; 2022202156@cqust.edu.cn (W.L.); zhouxinyu2914@126.com (X.Z.); 2023202068@cqust.edu.cn (X.C.); suguiyang2024@126.com (G.S.); 2 College of Aerospace Engineering, Chongqing University, Chongqing 400030, China

**Keywords:** thermoplastic composite, resistance welding, heat element, surface treatment

## Abstract

Thermoplastic composites are gaining widespread application in aerospace and other industries due to their superior durability, excellent damage resistance, and recyclability compared to thermosetting materials. This study aims to enhance the lap shear strength (LSS) of resistance-welded GF/PP (glass fiber-reinforced polypropylene) thermoplastic composites by modifying stainless steel mesh (SSM) heating elements using a silane coupling agent. The influence of oxidation temperature, solvent properties, and solution pH on the LSS of the welded joints was systematically evaluated. Furthermore, scanning electron microscopy (SEM) was utilized to investigate the SSM surface and assess improvements in interfacial adhesion. The findings indicate that surface treatment promotes increased resin infiltration into the SSM, thereby enhancing the LSS of the resistance-welded joints. Treatment under optimal conditions (500 °C, ethanol solvent, and pH 11) improved LSS by 27.2% compared to untreated joints.

## 1. Introduction

Advanced composites are widely used in aerospace, marine, and transportation due to their lightweight, high strength, and designability [1,2,3,4]. Compared with thermoset composites, thermoplastic composites possess superior strength, stiffness, toughness, damage tolerance, and impact resistance [5,6,7]. In addition, thermoplastic composites can be melted and re-cured multiple times for recyclability [8,9,10,11,12]. The joint part as the weak link of advanced composite structures, and therefore, how to realize the effective joint of structural components, becomes a key issue to realize the practical application of composites. At present, the main composite joint methods include traditional mechanical joint, adhesive, and fusion bond.

Conventional mechanical joints, although capable of transmitting large loads and being removable, are less efficient and prone to stress concentrations [8]. Adhesive bonding has a high process impact, long curing time, and low strength [13,14]. Fusion bonding, by heating the resin to the viscous flow state and then applying pressure cooling to form a welded joint, overcomes the shortcomings of the traditional mechanical bonding and glued joints, improving the reliability and stability of the joints, with a broader space for development [15]. Among them, resistance welding has the advantages of simple device, easy operation, low cost, high joint strength, and high competitiveness in rapid joining [16,17].

SSM (stainless steel mesh) is an efficient heat element used in resistance welding of thermoplastic composite structures. In such structures, the interfacial adhesion between the heated SSM and the resin directly affects the load-bearing capacity of the composite member [18,19,20,21]. Koutras et al. [18] and Dubé et al. [20] reported interlaminar damage modes for resistance-welded GF/PPS (Glass Fiber-Reinforced Polyphenylene Sulfide) and GF/PEI (Glass Fiber-Reinforced Polyetherimide) composites, stating that the damage occurs exclusively due to the nature of the resin adhesion of the composite itself. Interfacial adhesion between resin and SSM is often improved by modifying the substrate surface. Common surface treatments include sandblasting, mechanical grinding, chemical oxidation, etching, plasma treatment, and coupling agent treatment. Among them, silane coupling agent treatment is an effective surface modification method [22,23,24].

Silane coupling agents are widely used to enhance adhesion at metal–polymer interfaces, particularly in resistance welding, where they have been proven to be an effective surface modification technique. Recent studies have shown that silane grafting can significantly improve the performance of resistance-welded joints. For instance, Rohart et al. [25] demonstrated that silane coatings enhanced the adhesion between PPS and SSM, resulting in a substantial increase in lap shear strength (LSS) and failure load in carbon fiber-reinforced PPS (CF/PPS) resistance-welded joints. However, these techniques have not yet been applied to improve the interfacial bonding performance of GF/PP resistance-welded joints. Additionally, Ghosh et al. [26] found that silane hydroxyl groups can form hydrogen bonds with hydroxyl groups on metal surfaces and, through silanol groups, create covalent bonds with the substrate, leading to a stable siloxane network. This network not only enhances interfacial adhesion but also increases surface roughness [27,28]. In this study, aminopropyltriethoxysilane (APTES) was selected as the silane coupling agent to treat the surface of GF/PP composite resistance-welded joints, with the aim of improving their interfacial bonding performance.

While the fundamental mechanism of silane coatings is widely recognized, there is still no consensus on the specific factors that influence their adhesion performance. Factors such as the properties of the treated surface, the hydrolysis state of the silane, and the solution pH can all significantly affect the final adhesion outcomes of silane coatings. For example, Foerster et al. [29] demonstrated that acidic conditions result in stronger adhesion at the metal–polymer interface, whereas Rohart et al. [25] found that adhesion improved under alkaline conditions. Similarly, Golaz et al. [30] indicated that adhesion strength is enhanced in alkaline environments, although their study used water as the solvent, while Foerster et al. [29] used ethanol. Regarding oxidation temperature, Rohart et al. [25] showed that optimal LSS was achieved at 500 °C, while Li et al. [24] found that 350 °C was sufficient to improve shear strength. Therefore, optimizing the coating conditions for the silane coupling agent, including oxidation temperature, solvent type, and pH, is crucial for enhancing interfacial adhesion performance.

Although extensive research has investigated the adhesion performance at the metal–polymer interface in thermoplastic composites, the analysis on how different surface treatment methods and process parameters affect the performance of welded joints under mechanical loading is relatively limited. In this study, APTES was used to graft a silane coating onto the surface of the SSM, and the welding was performed using custom-developed resistance-welding equipment. The surface morphology of the SSM was observed using scanning electron microscopy (SEM), and LSS tests were conducted on both untreated and surface-treated SSM. Subsequently, the fracture morphologies of the untreated welded joints and those treated with different surface treatment parameter combinations were examined and compared, analyzing the effects of various process parameters on the failure modes of the welded joints.

## 2. Materials and Methods

### 2.1. Materials and Experimental Setup

**Figure 1:**
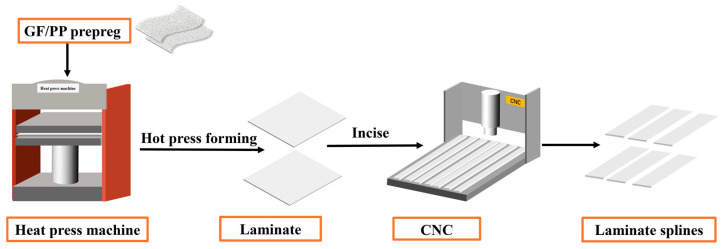
Schematic diagram of the preparation process of continuous glass fiber-reinforced polypropylene (GF/PP) specimens.

The GF/PP composite laminate is created by using the KS6010 GF/PP prepreg material [21] provided by Guangzhou Kingfa company (Guangzhou, China) and applying heat and pressure using a HBSCR hot press machine from Qingdao Huabo. The hot-pressing technique comprises the subsequent steps: Initially, the first part of the hot press machine is adjusted to a contact pressure of 0.2 MPa. The temperature is then raised to 160 °C and maintained for a duration of 26 min. Afterwards, the pressure is raised to 1 MPa, and the temperature is increased to 190 °C and maintained for a duration of 12 min. Next, while keeping the temperature constant, the pressure is raised to 2 MPa and maintained for a duration of 10 min. Ultimately, the pressure is decreased to 1 MPa, and the temperature is cooled to around 50 °C. The laminated board in this investigation consists of 8 layers, placed at a stacking angle of [0°/90°]2S. According to ASTM D5868 standard [31], the laminate is precisely cut into standard lap shear specimens measuring 100 mm × 25 mm × 2.4 mm using a CNC4060 engraving machine from Dongguan Jingyan Instruments Company (Dongguan, China). Figure 1 illustrates the method of preparing the laminates and the heating element (HE). The HE employed the 40-mesh SSM. After the hot-pressing process with the same process parameter with composite laminate, a 90 mm × 12.5 mm strip was cut. Except for the central 12.5 mm × 25 mm region of the HE, the other parts need to be removed by pre-flaming.

### 2.2. Design of Experiment: Taguchi Method

Table 1 lists 11 coating process parameters that have an impact on HE treatment. This study chose to use a 0.2 M concentration of sulfuric acid (H_2_SO_4_) to clean the surface of the HE, based on the experimental results of Hermas et al. [32]. The concentration of 0.2 M was selected due to the direct observation of the oxide layer undergoing active dissolution. The etching process was conducted at a temperature of 60 °C for a period of 30 min. Additionally, etching was selected as the preferred method over polishing due to the intricate mesh-like composition of SSM HE, which cannot be readily polished. Moreover, the length of oxidation significantly affects the level of oxide concentration. Hoikkanen et al. [33,34] determined the oxidation temperatures to be 350 °C and 500 °C, respectively. Furthermore, they observed that the oxide layer on the surface of the SSM HE exhibited an uneven condition following 5 min of oxidation, and the adhesive strength between the SSM and the resin would diminish after 100 min. Consequently, the experiment selected the median value of these two oxidation times, specifically 30 min. The efficacy of silane coatings’ grafting is contingent upon the solvent used for silane hydrolysis and the pH level of the solution. As a result, this study additionally examined these two factors. Solutions of silane with pH values of 5 and 11 were utilized, with water and ethanol selected as solvents as recommended by [33]. It is important to mention that even when ethanol was utilized, a specific quantity of water (5% by volume) had to be included due to the essential role of water in the hydrolysis of silanes. The duration for the hydrolysis of silane was set to 60 min. After considering the research of Foerster et al. [29], a silanization period of 60 min and a silane concentration of 2 vol% were chosen.

Using the Taguchi Design of Experiments (DoE) method, we conducted a detailed statistical analysis of the experimental results to determine the effects and significance of temperature, solvent type, and pH on the shear strength of welded joints. The statistical results are presented in Table 2. According to the one-way analysis of variance (ANOVA), temperature, solvent, and pH have significant effects on shear strength. Overall, effects of temperature and solvent are more particularly significant, while the effect of pH is relatively smaller but statistically significant. These findings suggest that in processing the resistance-welding process for GF/PP composites and stainless steel heating elements, temperature and solvent type should be prioritized as critical parameters.

Table 3 presents the configuration of three coating process parameters for SSM treatment. This study quantitatively analyzes the effects of oxidation temperature, solvent type, and solution pH on the adhesion between GF/PP and SSM. The goal is to determine the best coating parameters for obtaining excellent mechanical performance. Based on the chosen parameters, the overall length of the coating process should fall within the range of 105 to 165 min.

### 2.3. SSM Surface Treatment

**Figure 2:**
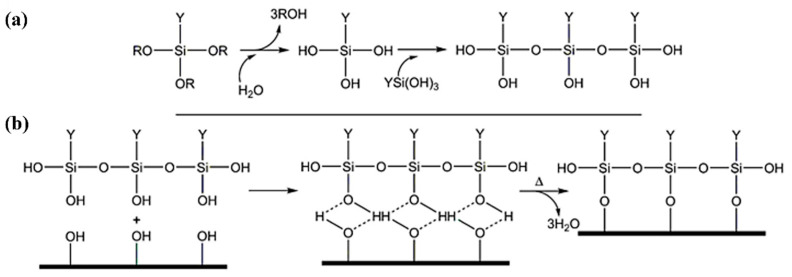
Silane hydrolysis mechanism (**a**) and condensation on an inorganic surface with crosslinking at high temperature (**b**). The metal surface represented as a bold line is generally pre-treated by acid and oxidized to increase the hydroxyl groups (-OH) concentration [25].

**Figure 3:**
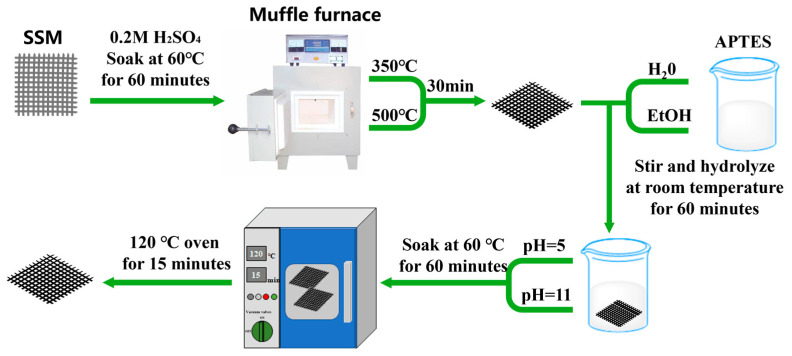
SSM surface treatment process.

The text utilizes SSM as a resistance-welding heat element; the silane molecules react with the oxides of the surfaces according to the mechanism shown in Figure 2. Alkoxyl groups are first hydrolyzed, either in solution or at ambient humidity. The newly formed hydroxyl groups can condense with the hydroxyl groups of neighboring molecules to form oligomers. The oligomers then condense on the surface and are processed by hydrogen bonding. Finally, covalent bonds are formed between the siloxane network and the surface by thermal crosslinking [25]. The process of surface treatment for SSM using a silane coupling agent involves multiple sequential steps in accordance with the steps described in Figure 3: Firstly, the process involves employing a 0.2 M H_2_SO_4_ solution in water at a temperature of 60 °C to corrode and eliminate impurities and oxides from the surface. This is followed by subjecting the material to oxidation in a muffle furnace for a duration of 30 min. Afterwards, the process of silane hydrolysis is carried out using a solution containing 2 vol% of APTES. The mixture is stirred at room temperature for a duration of 60 min. The SSM is submerged in APTES and incubated at a temperature of 60 °C for a duration of 60 min. To obtain coatings in acidic circumstances, the pH can be adjusted using acetic acid. Finally, the processed SSM is exposed to a 120 °C oven for a duration of 15 min.

### 2.4. Resistance-Welding Joint

**Figure 4:**
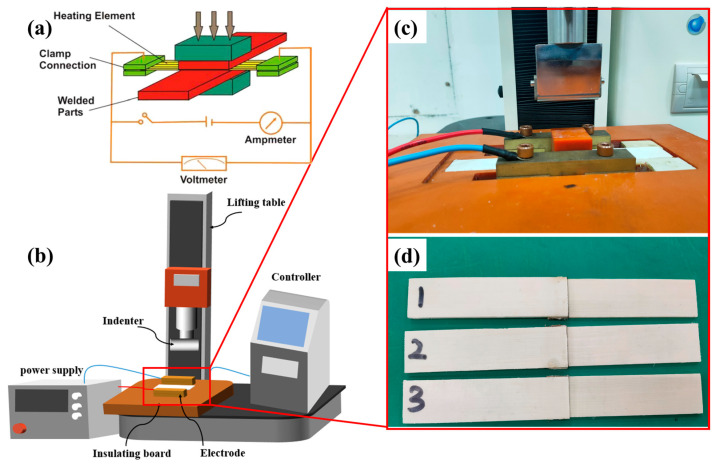
(**a**) Schematic diagram of resistance welding of composite materials [36]; (**b**) schematic diagram of welding platform; (**c**) local diagram of welding area; and (**d**) Single lap joint specimens 1-3 with the same welding parameters.

Figure 4a demonstrates the fundamental concept of resistance welding. The resistance-welding setup utilized in this experiment consists of multiple components, such as a direct current power source, electrodes, controller, insulating board, and pressure device, as shown in Figure 4b. An adjustable DC controlled power supply is used to provide the current, and brass electrodes are chosen for their excellent conductivity. The electrodes are linked to the power supply wires and the HE, producing thermal energy. The GF/PP layer strip designed for welding is placed between the electrodes, employing the slot in the insulating board. The HE is placed between the strip made of glass fiber-reinforced polypropylene (GF/PP) that needs to be welded. The part of the strip that is not covered is securely held by the brass electrode.

In the welding process, welding pressure is critical as it not only ensures sufficient contact between the welded surfaces but also promotes molecular diffusion. Insufficient pressure may result in voids at the joint, weakening the bonding strength, while excessive pressure can lead to excessive resin extrusion, fiber slippage, or interface deformation, thereby compromising welding quality. Welding time and current together determine the total input energy. If the power is too low and the time too short, energy may dissipate excessively, leading to insufficient temperature at the joint and preventing adequate material melting. Conversely, excessively high power over a long time may cause a large temperature gradient, resulting in uneven temperature distribution, material degradation at the edges, and incomplete melting in the center, all of which can weaken joint strength [15,27,35]. The optimized welding parameters were determined as follows: welding pressure of 15.6 N, current flow time of 1.2 min, and input current of 10.6 A. After the welding operation, a 30 s pressure cooling was conducted, and the welded resistance strips are shown in Figure 4d. The average thickness of the thermoplastic surface layer in the welding area of the resistance-welded single-lap joint specimen was measured to be 0.90 mm [19]. In addition, single-lap benchmark specimens were also fabricated for comparison.

### 2.5. Resistance-Welding Joint LSS Test

**Figure 5:**
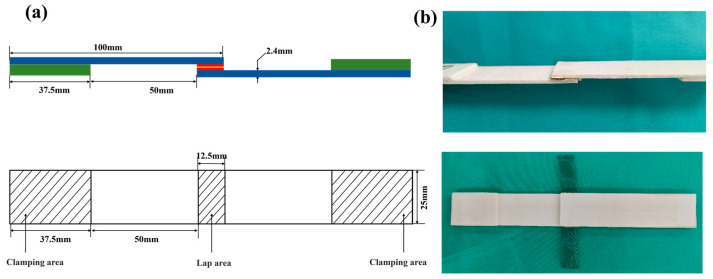
Resistance-welding joint (**a**) structure size drawing; and (**b**) specimen topography drawing.

**Figure 6:**
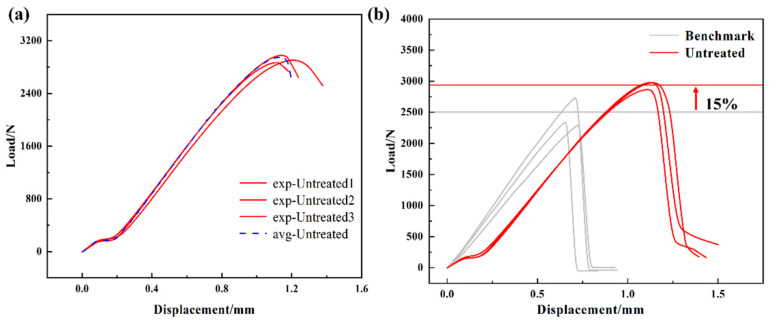
(**a**) Untreated control group; (**b**) comparison of the shear performance of resistance-welding specimens and benchmark specimens.

**Figure 7:**
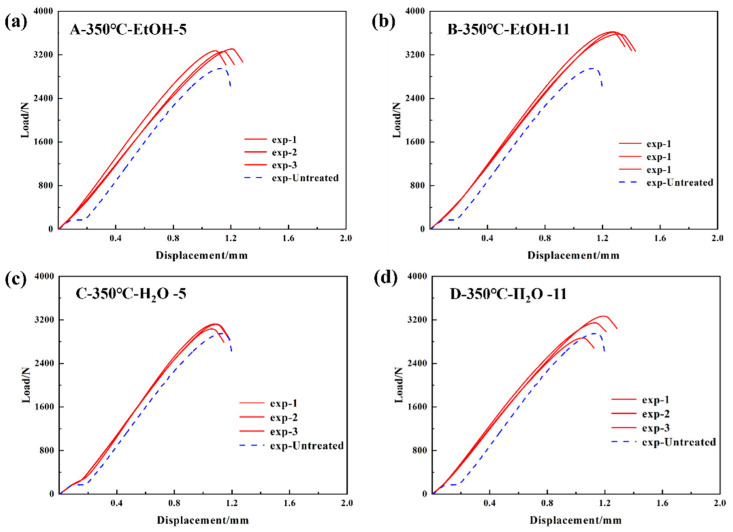
Load–displacement curves of GF/PP lap joints at 350 °C oxidation temperature (**a**) A-350 °C-EtOH-5; (**b**) B-350 °C-EtOH-11; (**c**) C-350 °C-H_2_O-5; and (**d**) D-350 °C-H_2_O-11.

**Figure 8:**
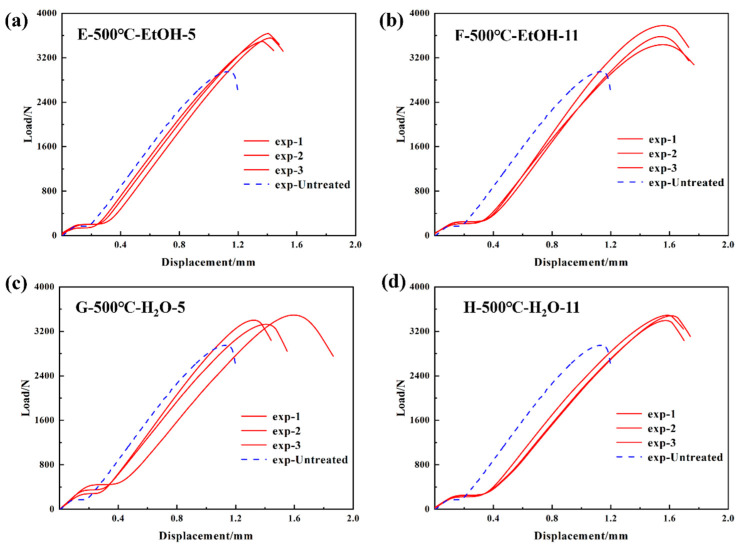
Load–displacement curves of GF/PP lap joints at 500 °C oxidation temperature (**a**) E-500 °C-EtOH-5; (**b**) F-500 °C-EtOH-11; (**c**) G-500 °C-H_2_O-5; and (**d**) H-500 °C-H_2_O-11.

To prevent any relative sliding between the specimen and fixtures, as well as to prevent any local stress concentration and early failure of the specimen, reinforcing patches were bonded to both ends of the specimen. The reinforcing patch used was a GF/PP laminate measuring 37.5 mm × 25 mm × 2.4 mm. The parameters of the resistance-welding head structure are illustrated in Figure 5a. Afterwards, the GF/PP resistance-welding head was subjected to LSS testing utilizing the UTM21604 electronic universal testing machine. The morphology of the specimens is depicted in Figure 5b. To ensure the reliability of the experimental results, each group of experiments was repeated at least three times. For the untreated control group, the data were averaged to generate a representative mean curve for the untreated specimens as shown in Figure 6a. The mean curve for the untreated group is depicted as a blue dashed line in Figure 7 and Figure 8.

After calibrating the test machine, the specimen was firmly fastened between the upper and lower grips of the testing machine, ensuring that the centerline of the specimen was aligned with the centerline of the testing machine. Afterwards, load was exerted at a velocity of 2 mm/min. During the tensile process, the load–displacement curve was continuously monitored in real-time. If there was a sudden decrease in the load or if the specimen failed, the loading process was stopped, the grips were released, the test data were recorded, and the failure of the specimen was seen. The LSS of the resistance-welding head was determined by Formula (1), and subsequently, the experimental data were examined.

In the context of the formula: *τ* represents tensile shear strength (N/mm^2^); *F_max_* denotes maximum tensile force (N); *L* stands for lap length (mm); and *b* represents lap width (mm).

(1) $$\tau =\frac{F_{max}}{Lb}$$

### 2.6. The Characterization of Failure Modes

This study evaluated the bonding strength of thermoplastic composite material resistance-welding units and resin from both microscopic and macroscopic viewpoints. The surface morphology of SSM was examined at the micro level using a SEM both before and after applying different parameter combinations. The primary objective was to examine alterations in surface morphology and investigate the impact of SSM surface morphology on the adhesion of thermoplastic composite material resistance-welding units and resin. A characterization study was performed on the broken interface of specimens after LSS testing to collect pertinent data on interface bonding strength at a macro level.

## 3. Results and Discussion

### 3.1. Tensile Properties

In this work, tensile tests were performed on resistance-welded heads treated with various combinations of parameters. The load–displacement curves of the resistance-welded heads were then displayed. The control group consisted of a resistance-welded head made from SSM without any surface treatment. The average maximum tensile load for this group was 2875 N, as shown in Figure 6a. The untreated specimens were compared with the benchmark specimens, as shown in Figure 6b. The results show the untreated specimens’ maximum load was significantly higher, with a 13% difference between the resistance-welded (2875 N) and benchmark (2502 N) specimens.

At an oxidation temperature of 350 °C, ethanol and water are employed as solvents, while the pH of the solution is adjusted to 5 and 11. These values correspond to the resistance-welding heads that have undergone treatment using parameter combinations A–D, as specified in Table 3. Figure 7 displays the load–displacement curves of resistance-welded joints that were subjected to various parameter combinations during tensile testing. It reveals that the maximum tensile loads of resistance-welded joints exceeded those of the control group. Figure 7b shows that GF/PP resistance-welded joints, under specific conditions (350 °C oxidation temperature, ethanol solvent, pH 11), achieved a 26.1% higher maximum tensile load (3625 N) than untreated joints.

At an oxidation temperature of 500 °C, modifications in the characteristics of the solvent and the pH of the solution correspond to the parameter combinations E–H in Table 3 for the treatment of the resistance-welded unit. After subjecting the resistance-welded joints to each combination of parameters, the load–displacement curves from the tensile tests are shown in Figure 8. It demonstrates that the maximum tensile load of all resistance-welded joints exceeding that of the control group. Figure 8b presents the optimal parameter combination (500 °C oxidation, ethanol solvent, pH 11), yielding a GF/PP resistance-welded joint with a peak tensile load (3656 N) 27.2% higher than the untreated joint.

**Figure 9:**
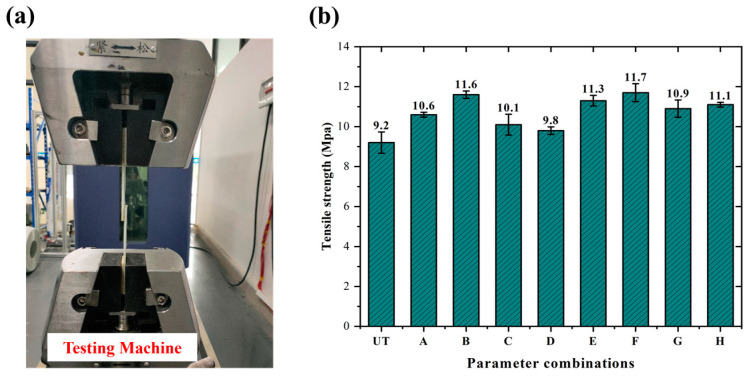
(**a**) Diagram of the lap shear experimental setup; (**b**) comparison of tensile strength of different parameter combinations.

At the initial loading stage, the load–displacement curves for all treatment combinations (A–H) exhibited a consistent linear increase, indicating that the welded-joint materials underwent elastic deformation during the early phase of tensile testing. Compared to the untreated specimens (represented by the blue dashed line), the treated specimens demonstrated higher peak load. As the load increased, the curves transitioned from linear to nonlinear due to the deformation or damage at the welding area. After reaching the peak load, the curves dropped sharply due to fracture. The LSS values of the resistance-welded joints are determined by applying different parameter combinations and calculating them using Formula (1), which is based on the maximum tensile load. The configuration and results of the LSS test are illustrated in Figure 9a,b. The mean tensile strength of the untreated resistance-welded joint is 9.2 ± 0.5 MPa. The resistance-welded joint, which was treated with an oxidation temperature of 500 °C, ethanol as a solvent, and a solution pH of 11, shows a tensile strength of 11.7 ± 0.5 MPa. This is a 27.2% increase compared to the untreated joint.

### 3.2. Failure Modenb

#### 3.2.1. Micro-Interfacial Performance

**Figure 10:**
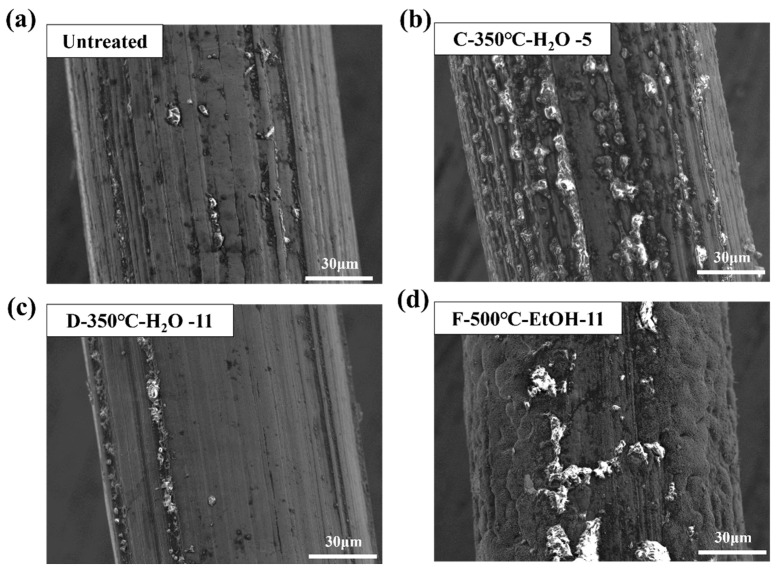
SSM surface topography (**a**) untreated; (**b**) C-350 °C-H_2_O-5; (**c**) D-350 °C-H_2_O-11; and (**d**) F-500 °C-EtOH-1.

The SEM analysis was conducted on the surface of SSM with no surface treatment, as well as three groups of SSM treated with different parameter combinations. These combinations include C (oxidation temperature of 350 °C, water as a solvent, solution pH 5); D (oxidation temperature of 350 °C, water as a solvent, solution pH 11); and F (oxidation temperature of 500 °C, ethanol as a solvent, solution pH 11). Figure 10 displays SEM images of SSM with various surface treatments. The untreated SSM surface exhibited a peaked and concave morphology with a more prominent groove-like structure. The surface morphology of the modified treated SSM underwent significant changes, characterized by a reduction in surface grooves and an increase in the presence of particles with diameters less than 1 mm, as compared to the untreated SSM. These observations suggest that the shallow grooves have been filled in and an oxide layer has been successfully formed on the surface. The parameter combination F treatment results in a surface concave–convex structure that is more uniform and detailed. This indicates that the surface grooves have been filled with particles, resulting in the formation of a denser oxide layer, as shown in Figure 10d, which benefits the GF/PP resistance-welding interfacial strength.

**Figure 11:**
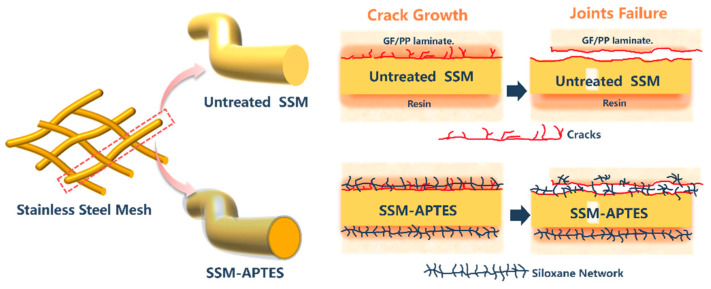
A schematic diagram illustrating the bonding mechanism at the interface of untreated single SSM GF/PP joints and silane-treated GF/PP resistance-welded joints. Adapted from X. Li, M. Sun, J. Song, T. Zhang, Y. Zhao, and K. Wang [24].

Figure 11 illustrates a comparison of crack initiation and propagation patterns in untreated SSM and silane-coupling-agent-treated SSM joints which is based on existing literature [24]. When treating the HE with a silane coupling agent, the polymer chains in the matrix become intertwined with the grafted functional groups on the surface of SSM. This happens when there is enough pressure at the processing temperature. As a result, an interfacial layer is formed on the surface, which consists of PP, a siloxane network, and an oxidized layer. This multicomponent interfacial layer is effective in altering the way cracks start and spread. During the LSS test, when the joint experienced higher shear loads, the intermingling of the PP and siloxane networks prevented the formation of cracks by absorbing significant localized energy and decreasing the concentration of stress around the crack tip. Furthermore, while crack propagation typically occurs along the SSM surface, the direction of propagation can change because of effective interlocking. This interlocking increases the energy barrier for crack propagation, causing the crack to not only conform to the SSM surface but also extend into the interstitial space of the molecular entanglement. Consequently, there is an increase in energy dissipation. In addition, some researchers have discovered that the strong adhesion between the SSM and the siloxane network enhances the performance of resistance-welding tips, as observed in the improvement of LSS [25].

#### 3.2.2. Fracture Analysis

**Figure 12:**
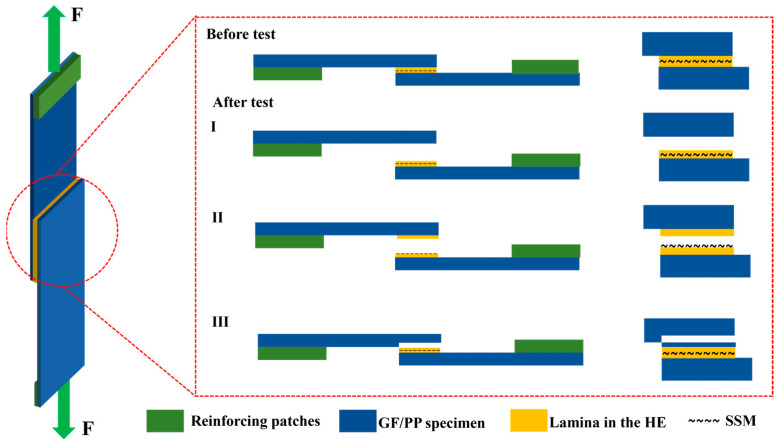
Typical failure mode Types I, II, and III of single-lap shear.

The study involved examining the visible characteristics of cross-sections and analyzing the appearance of fractures in resistance-welded joints. This was studied to understand how failure occurs and to evaluate how various factors affect the bonding of SSM-PP materials. Figure 12 shows that the surfaces of shear failure specimens exhibited three distinct failure mechanisms, classified as Type I, Type II, and Type III failures, illustrating the three common failure types observed. A Type I failure commonly occurs at the interface between the HE and the surface of the welded laminate, namely a bonding failure. The Type II failure mode involved the interlayer failure of the HE, which was defined by the detachment of the matrix from the SSM, thereby exposing it. A Type III failure refers to the failure of the material that was supposed to be bonded together, specifically the failure of the interlayer in GF/PP laminates.

**Figure 13:**
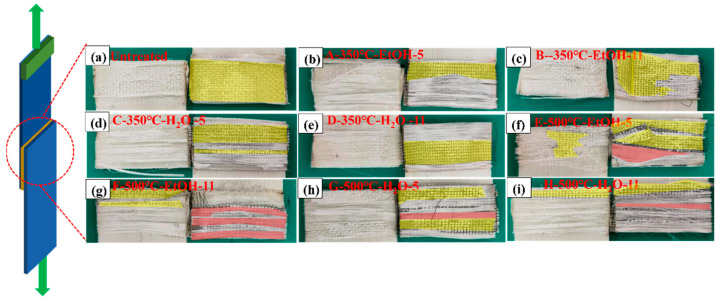
Typical failure fracture surfaces of resistance-welded heads (**a**) untreated; (**b**) A-350 °C-EtOH-5; (**c**) B-350 °C-EtOH-11; (**d**) C-350 °C-H_2_O-5; (**e**) D-350 °C-H_2_O-11; (**f**) E-500 °C-EtOH-5; (**g**) F-500 °C-EtOH-11; (**h**) G-500 °C-H_2_O-5; and (**i**) H-500 °C-H_2_O-11.

To solve the influence of the bonding mechanism on the LSS properties of GF/PP resistance joints, the fracture surfaces of resistance-welded heads after LSS testing were macroscopically analyzed in combination with the tensile strength comparison graphs of different parameter combinations in Figure 7 of Section 3.1. The fracture surfaces of resistance-welded joints treated with different parameters are shown in Figure 13. Figure 13a shows the fracture surface morphology of the GF/PP resistance-welded joint without surface treatment. Almost no residual polymer matrix remains on the SSM, with significant delamination occurring between the matrix and the SSM, indicating a Type II interlayer failure mode for the resistance-welded joint’s heating element (HE), as highlighted in yellow in Figure 13.

In the resistance-welded joints treated with the parameter combination A/B/C/D, the fracture surface exhibited an interlayer fracture in the HE as well as debonding between the HE and the laminate. Partial delamination of the matrix from the metal mesh and localized exposure of the mesh were observed, indicating a mixed failure mode comprising Type I and Type II failures. Compared to untreated joints, these welded joints demonstrated an increase in LSS.

Further examination of the fracture surfaces of joints treated with the parameter combination E/F/G/H revealed not only a mixed failure mode of Type I and Type II, but also the presence of a Type III failure mode (indicated in red in Figure 13), characterized by adhesive failure and interlaminar failure of the GF/PP laminate. The LSS test results showed a significant improvement over the joints treated with the ABCD combination. Additionally, the fracture surfaces of joints treated with parameter combinations E and F showed large areas of Type III failure, which resulted in the higher LSS.

**Figure 14:**
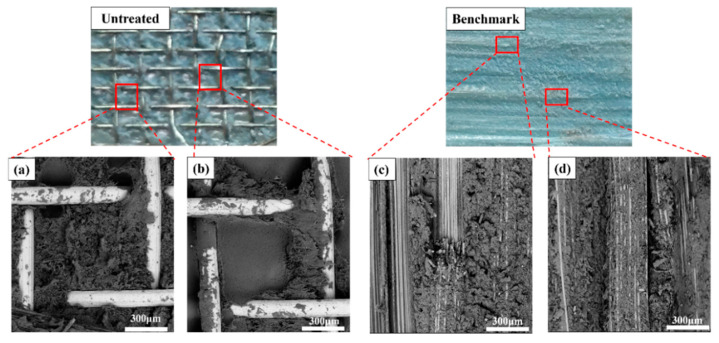
Fracture surfaces of welded joints after tensile testing of untreated HE and benchmark. (**a**,**b**) is the local micrographs for the untreated welded specimen, and (**c**,**d**) is the local micrographs for the benchmark specimen.

Figure 14 presents the micrographs of the fracture surfaces after tensile testing for the untreated welded specimen and the benchmark specimen. The left image shows the tensile fracture surface of the resistance-welded specimen. Figure 14a,b show that, although the resin partially penetrated the metal mesh and formed a good wettability during the welding process, significant interfacial detachment between the matrix and the SSM took place after the tensile test. It is shown by the separation of the polypropylene matrix from the SSM under tensile loading, with the metal mesh partially exposed, exhibiting a Type II interlayer failure mode. The right image demonstrates the tensile fracture surface of the hot-pressing comparison specimen, fiber breakage, and interlayer failure that were observed.

## 4. Conclusions

This study was to explore techniques for improving the adhesion performance of GF/PP resistance-welded joints. The study also investigates the influence of factors such as oxidizing temperature, solvent properties, and solution pH by LSS test. The investigation focused on studying the adhesion enhancement mechanism between resistance-welded units and the resin of thermoplastic composites by examining the macroscopic failure of resistance-welded joints and the microscopic surface of SSM. The following conclusions were made:

The load–displacement curves obtained from the LSS tests indicated that the resistance-welded heads made from the untreated SSM exhibited an average maximum tensile load of 2875 N. In contrast, the resistance-welded heads treated with an oxidation temperature of 500 °C, ethanol as a solvent, and a solution pH of 11 demonstrated an average maximum tensile load of 3656 N, representing the optimal LSS result of 11.7 ± 0.5 MPa. A significant improvement of 27.2% in tensile strength was observed when comparing the resistance-welded units with untreated SSM.

The surface of the untreated SSM has a distinct groove-like structure, as observed using SEM. The grooves on the surface of the SSM treated with an oxidation temperature of 350 °C, water as a solvent, and a solution pH of 5, are not very deep. On the other hand, the grooves on the surface of the SSM treated with an oxidation temperature of 500 °C, ethanol as a solvent, and a solution pH of 11, are barely noticeable. This suggests that the particles on the surface have filled in the grooves, and the oxidized layer has been effectively formed.

The macroscopic analysis of the fracture surface reveals a distinct alteration in the failure mode of the resistance-welded joint when a silane coupling agent is employed as the resistance-welding unit following SSM treatment. The major mode of failure transitions from interlayer failure Type II to a mix of interlayer failure and bonding failure Type I. The alteration is primarily attributed to the enhanced wetting characteristics of the SSM due to surface treatment, facilitating more efficient resin penetration into the pores of the SSM.

## Figures and Tables

**Table 1:** Coating influencing factors used for the Taguchi DoE.

Factor	Level − 1	Level + 1
Temperature (T_ox_)	350 °C	500 °C
pH	5	11
Solvent	EtOH	H_2_O

**Table 2:** Results of one-way analysis of variance (ANOVA) for shear strength of welded joints.

Source of Variation	SS	df	MS	F	*p*-Value
Temperature	685,956.7	1	685,956.7	215.476	0.00000163
Solvent	352.5006	1	352.5006	575.931	0.00000006
pH	33.35063	1	33.35063	6.920371	0.03388012

**Table 3:** Coating process factor design.

Parameters	T_ox_	Solvent	pH
A	350	EtOH	5
B	350	EtOH	11
C	350	H_2_O	5
D	350	H_2_O	11
E	500	EtOH	5
F	500	EtOH	11
G	500	H_2_O	5
H	500	H_2_O	11

## Data Availability

The original contributions presented in this study are included in the article, further inquiries can be directed to the corresponding authors.
